# Health workers’ and hospital administrators’ perspectives on mistreatment of women during facility-based childbirth: a multicenter qualitative study in Ghana

**DOI:** 10.1186/s12978-022-01372-3

**Published:** 2022-03-29

**Authors:** Kwame Adu-Bonsaffoh, Evelyn Tamma, Ernest Maya, Joshua P. Vogel, Özge Tunçalp, Meghan A. Bohren

**Affiliations:** 1 Department of Obstetrics and Gynaecology, University of Ghana Medical School, Accra, Ghana; 2 Holy Care Specialist Hospital, Accra, Ghana; 3 School of Public Health, University of Ghana, Accra, Ghana; 4 Maternal, Child and Adolescent Health Program, Burnet Institute, Melbourne, VIC Australia; 5 UNDP/UNFPA/UNICEF/WHO/World Bank Special Programme of Research, Development and Research Training in Human Reproduction (HRP), Department of Sexual and Reproductive Health and Research, World Health Organization, 1211 Geneva, Switzerland; 6 Gender and Women’s Health Unit, Centre for Health Equity, Melbourne School of Population and Global Health, The University of Melbourne, Carlton, VIC 3053 Australia

## Abstract

**Background:**

Globally, mistreatment of women during facility-based childbirth continues to impact negatively on the quality of maternal healthcare provision and utilization. The views of health workers are vital in achieving comprehensive understanding of mistreatment of women, and to design evidence-based interventions to prevent it. We explored the perspectives of health workers and hospital administrators on mistreatment of women during childbirth to identify opportunity for improvement in the quality of maternal care in health facilities.

**Methods:**

A qualitative study comprising in-depth interviews (IDIs) with 24 health workers and hospital administrators was conducted in two major towns (Koforidua and Nsawam) in the Eastern region of Ghana. The study was part of a formative mixed-methods project to develop an evidence-based definition, identification criteria and two tools for measuring mistreatment of women in facilities during childbirth. Data analysis was undertaken based on thematic content via the inductive analytic framework approach, using Nvivo version 12.6.0.

**Result:**

Health workers and hospital administrators reported mixed feelings regarding the quality of care women receive. Almost all respondents were aware of mistreatment occurring during childbirth, describing physical and verbal abuse and denial of preferred birthing positions and companionship. Rationalizations for mistreatment included limited staff capacity, high workload, perceptions of women’s non-compliance and their attitudes towards staff. Health workers had mixed responses regarding the acceptability of mistreatment of women, although most argued against it. Increasing staff strength, number of health facilities, refresher training for health workers and adequate education of women about pregnancy and childbirth were suggestions to minimize such mistreatment.

**Conclusion:**

Health workers indicated that some women are mistreated during birth in the study sites and provided various rationalizations for why this occurred. There is urgent need to motivate, retrain or otherwise encourage health workers to prevent mistreatment of women and promote respectful maternity care. Further research on implementation of evidence-based interventions could help mitigate mistreatment of women in health facilities.

**Supplementary Information:**

The online version contains supplementary material available at 10.1186/s12978-022-01372-3.

## Plain language summary

Respectful maternity care is vital to achieving positive pregnancy and childbirth experiences for women and their families. Mistreatment of women during childbirth at facilities can negatively impact women’s future health seeking behaviors and utilization of maternal care services. The experiences and perspectives of doctors, midwives and nurses working in labour wards are vital in understanding how women are treated during childbirth, and what measures can be taken to prevent it. In this study, we explored the opinions of health workers and hospital administrators on how women are treated during childbirth to determine the gaps in the quality of maternal care in health facilities in Ghana.

Participants expressed mixed feelings concerning mistreatment of women during childbirth. Most were aware of the occurrence of mistreatment in health facilities including physical and verbal abuse, and denial of preferred position for childbirth and companionship. The reasons provided for mistreatment included low staff capacity, high workload, non-compliance by women and poor attitudes towards health workers. Most health workers were against mistreatment during childbirth. Participants thought mistreatment could be minimized by improving staff skills, refresher training, and childbirth preparation education for women. Our study indicates the need to motivate, retrain or encourage health professionals to provide respectful care to women during childbirth to improve their experience of care. Further research to help implement better maternity care devoid of mistreatment in health facilities in Ghana is needed.

**Supplementary Information:**

The online version contains supplementary material available at 10.1186/s12978-022-01372-3.

## Introduction

Globally, mistreatment of women during facility-based childbirth continues to impact negatively on the quality of maternal healthcare provision and utilization. Mistreatment remains a global public health challenge, considering its prevalence, associated infringements on women’s rights, and adverse impacts on health and well-being of the affected women and families [1–3]. In a recent World Health Organization (WHO) multi-country study comprising both labor observation and community survey, about 4 in 10 women experienced some form of mistreatment including physical, verbal, discrimination and neglect during facility-based childbirth [4]. Women who experience mistreatment in healthcare facilities, particularly in limited-resource settings, may resort to alternative services for childbirth such as traditional health attendants, where they feel better treated and respected [5, 6].

In Ghana, approximately 90% of pregnant women receive four or more antenatal visits; the rate of facility births is about 80%, increasing from 54% over the past decade [7]. Although this increase in maternal healthcare service utilization is positive, maternal mortality ratio has improved only marginally, from 371 in 2005 to 308 maternal deaths per 100,00 livebirths in 2017 [8]. This disconnect between improved healthcare access and persistent high levels of maternal mortality suggests the quality of maternity care services is suboptimal [9]. Promisingly, recent evidence indicates that promoting respectful maternity care (RMC) can improve maternal health care utilization [2, 10].

The negative impact of mistreatment of women in health facilities has been recognized by the global community and active efforts have been implemented to minimize its occurrence. WHO defines respectful maternity care as the care organized for and provided to all women in a manner that maintains their dignity, privacy and confidentiality, ensures freedom from harm and mistreatment, and enables informed choice and continuous support during labor and childbirth [11]. The types of mistreatments during childbirth that have been identified include physical, verbal, and sexual abuse, stigma and discrimination, neglect, substandard care, poor communication and lack of privacy and confidentiality [3, 12]. WHO’s 2018 intrapartum care recommendations for a positive childbirth experience emphasize the importance of respectful, supportive care interventions for women giving birth, as well as the need for effective, culturally sensitive communication with women and their families [11].

In a previous qualitative paper, we reported the occurrence of major types of mistreatments experienced by women in Ghana including verbal abuse (shouting, insults), physical abuse (pinching, slapping) and neglect [6]. Similar reports indicate that, mistreatment and disrespectful care are pervasive in the country [5, 13, 14]. These events were more prevalent during the second stage of labor, and amongst adolescent mothers [4, 6]. The views of health workers are vital to achieving a comprehensive understanding of the phenomenon, especially as health workers’ behaviors and health system failures have been implicated in some forms of mistreatment [3, 5, 15–17]. However, there is insufficient evidence on the perspectives of maternity care providers and administrators in Ghana on this issue. The objective of this article was to explore the perspectives of health workers and hospital administrators on mistreatment of women during childbirth in Ghana, to identify opportunities for improving the quality of maternal care. This qualitative study, provided evidence for the development and validation of two tools for measuring mistreatment of women during childbirth at health facilities.

## Methods

### Study design and sites

This was a qualitative phenomenological study conducted between May to July 2015 in two major towns (Koforidua and Nsawam) in the Eastern region of Ghana. The study was part of formative research to develop an evidence-based definition, identification criteria and two tools for measuring mistreatment of women during childbirth in health facilities. The study protocol has been published previously [18]. We have also published our findings on women’s perspectives on mistreatment during childbirth in Ghana [6]. In this article, we present the perspectives of health workers (including nurses, midwives and doctors) and hospital administrators, to complement the analysis of women’s perspectives and inform development of quality improvement strategies to enhance respectful maternity care in the country.

The two study sites comprised secondary level facility or higher, one facility located in a rural or peri-urban area and the other urban with well-defined catchment area. To be eligible, the included health facility required a minimum of 3 doctors/specialists, 3 nurses/midwives working in the maternity unit of the hospital and at least 2 administrative personnel in the facility. The two study sites (Koforidua and Nsawam) are located in the same administrative region in Ghana with a population of approximately 2.6 million [19]. Koforidua, the capital of the Eastern region of Ghana, is an urban location with a large regional hospital including a specialist maternity care unit. Comparatively, Nsawam is a peri-urban city with a district hospital which also provides maternity care services. In the Eastern region, about 97% of pregnant women receive antenatal care services from skilled providers and about 77% give birth at health facilities with nearly 80% supervision from skilled birth attendants [7].

### Study participants and sampling

We used in-depth interviews (IDIs) to obtain comprehensive knowledge on how women are treated during facility-based childbirth from health workers (Additional file 1: Appendix 1) and hospital administrators (Additional file 2: Appendix 2). The use of IDIs is considered one of the most powerful methods in gaining comprehensive understanding of people including personal experiences and perspectives and exploring relevant topics in depth [20]. Purposive sampling was employed to recruit the participants from their places of employment. The types of study participants included nurses/midwives, medical officers (doctors), and health administrators with different levels of experiences (e.g. both junior and senior staff).

### Data collection

A total of twelve research assistants were trained to conduct the interviews, with six per study site. All research assistants were non-clinical health researchers, and had at least a bachelor’s degree education, with experience in conducting qualitative interviews for research. Prior to the data collection, a training workshop was organized for all the research assistants, field supervisor and all the members of the research team and including training on study procedures to ensure consistency. During the training workshop, a mock interviewing exercise was organized for the research assistants to ensure consistency in the procedures for the data collection in both English and Twi (a local language). The interview guide was developed based on a systematic review of mistreatment of women during childbirth [3] and adapted after the pilot interviews.

Each interview was facilitated by two experienced research assistants, one who acted as a moderator and one as a note-taker. There was no prior relationship between the participants and interviewers, and no contact with the participants after the interviews were completed. We conducted all interviews in either English or Twi and audio recorded. After each interview, immediate debriefing was carried out by the research assistants to document the comments and observation that emerged during the interview with incorporation of the field notes. On the average, each interview lasted between 45 to 60 min and all the interviews were undertaken in a private room at the participating hospitals. Recruitment of participants continued until no new themes emerged from the data (data saturation reached).

### Data management and analysis

The interviewers transcribed all the audio recordings in English immediately after the interviews. The interviews conducted in Twi (a local language) were directly transcribed into English by the interviewers, shortly following the interviews. The completeness and accuracy of the transcripts were cross-checked and confirmed by the social scientist and field supervisor in the research team. Prior to the data analysis, three-day workshop on qualitative data synthesis and analysis was organized and facilitated by our collaborating research team from WHO. The participants of the workshop included the research assistants and investigators comprising social scientists, public health physicians, obstetricians and the field supervisor as well the WHO research team. The inclusion of different investigators with relevant specialties provided appropriate reflexivity and improved the interpretation of the study findings. Reflexivity describes the researchers background (preconceptions) and positions which can influence the research in terms of the research question, data collection method, analysis, interpretation and communication of the findings [21].

In this study, the codebook for the analysis was developed by KAB and ET following identification of the major themes that emerged from iteratively reading the transcripts. This was necessary to ensure comprehensive familiarization with the transcripts and to appreciate the worldview of the respondents in terms of their individual perceptions of mistreatment of women during childbirth. Discrepancies in the coding process were discussed between KAB and ET until consensus was reached. The generated codes based on the major themes identified in the transcripts were then transferred into NVivo (QSR International (1999) NVivo Qualitative Data Analysis Software, Version 12.6.0 [Software]) which was used for the thematic analysis. The main qualitative analytic framework used in this study was the inductive approach which involves repeated reading of the transcripts resulting in the identification of the common themes discussed by the respondents [22]. Triangulation of the results was achieved by the case-mix involving the different health workers (nurses, midwives and doctors of varied experiences) and the hospital administrators from two study sites of different catchment areas (data source triangulation) [20]. Also, multiple research assistants and note takers (investigator triangulation) conducted the interviews and coding of the transcripts was undertaken by two different researchers. We validated the themes and results of the study with the research assistants and the note takers during the analysis workshop but not the study participants. This paper was reported according to the Consolidated criteria for reporting qualitative research (COREQ) [23].

## Results

Thirty participants were invited to participate in the in-depth interviews, of which six declined due to their unavailability resulting in a total of 24 participants. Table 1 describes the sociodemographic characteristics of participants. In total, 9 midwives/nurses, 11 doctors and 4 administrators were interviewed. Majority of the respondents (18) were between the ages of 25–39 years, single (12) and female (14). Most respondents had been working in their respective health facilities for a period of 1–4 years.

**Table 1:** Sociodemographic characteristics of participants: health workers

	Nurse/midwives n = 9	Doctors n = 11	Administrators n = 4
Age (years)			
30–39	6	11	1
40 +	3	–	3
Marital status			
Single	3	5	4
Married	4	6	–
Widowed	2	–	–
Gender			
Female	9	3	2
Male	–	8	2
Years of practice at facility			
1–4	6	9	3
5–9	1	1	1
10 +	2	1	–
Hospital			
Tertiary facility	5	7	1
Secondary facility	4	4	3

The major themes on mistreatment during childbirth that emerged from the IDIs include the following:

1. General perception of women’s satisfaction during childbirth
2. Support systems for women during childbirth
3. Occurrence of mistreatment of women during childbirth
4. Perceived factors that influence mistreatment during childbirth
5. Acceptability or rationalization of mistreatment during childbirth
6. Major challenges faced by health workers

### General perception of women’s satisfaction during childbirth

Health workers had diverse responses regarding women’s satisfaction with the childbirth services provided at their facilities. Some believed that women were satisfied with their care during birth, while others considered women’s satisfaction as variable, depending on their experiences. Some respondents indicated that women who experienced standard quality of care were usually satisfied.

“I think that it’s a two-way thing. Some of them think that …[they were] shouted at…that we actually don’t give off our best. But there are some of them who also think …they really took very good care of me and I know what was going on. Some of them think that we are doing very well” (27 years, Medical officer).

“In general, I think they are treated well…. Childbirth is not easy, the pain alone can make someone forget that they are in labour or something and they use harsh words, so if you are not calm you may also use the same harsh words on them” (26 years, Midwife).

Health workers described their labour wards as busy environments. Some women travel longer distances to utilize their maternity services. The providers believed that women’s willingness to travel long distances to access care was due to the high quality of care they provided compared to other facilities at lower levels of care.

“In our health facility I must confess that they are treated very well, but I wouldn’t say that our midwives or staffs are angels. They are not, but with continuous quality assurance training, they do their best to make the women feel comfortable and that is why we get a lot of cases here. We get a lot of cases coming in from even Accra which is not our catchment area” (34 years, Medical officer).

### Support systems for women during childbirth

Health workers described support to women during labour including physical, emotional and psychological elements. It included words of encouragement and gentle massage from providers, family or friends. The presence of a birth companion was identified as a way to relieve women’s stress. Some respondents considered anxiety to be more common amongst women who had not given birth before, and that these women, in particular, need supportive care during childbirth.

“Support comes in a number of ways, we have the physical, we have the emotional and we have the psychological. For the patients who haven’t delivered before, they don’t even know how it is like to be in labour and go through the pain and deliver. They are also anxious. They will need a lot of support and that is how we come in to support them so with encouragement, trying to reduce their pain and then making them as comfortable as they can” (34 years, Medical officer).

“I think for labour support, one, for the woman having her relatives around either the husband, mother or sister, whoever she wants to be around is the number one way the woman feels supported because she feels she is not alone, she has somebody to lean on. Many women want to be pampered, want to be caressed, so at that time that she is in distress and there is somebody there who she thinks best understands her, I think it’s best for her.”. (32 years, Medical officer).

### Occurrence of mistreatment of women during childbirth

Almost all participants had heard of women being mistreated during childbirth. They described instances of physical, verbal and emotional abuse by clinical staff towards women, as well as instances of denying women birth companions or preferred birthing position.

#### Physical abuse

Some respondents described situations where women were physically hit when they were unable to push during birth. Some health workers described instances where women were physically held down strongly with their legs apart, to prevent them from closing their legs when the baby was crowning, ostensibly to prevent birth asphyxia.

“Mostly, it’s during the pushing time that those things happen because everyone is like very aggressive to just get the baby out. So sometimes it may happen that someone lashed her” (27 years, Medical officer).

#### Verbal abuse

The use of abusive words or shouting at women was described as a common occurrence, especially when women were in the second stage of labour. Health  workers described instances where women are screamed at to facilitate birth of the baby, and instances where women were shouting at for not following instructions from clinical staff.

“The midwife was shouting at her. They ask her to lie down and she says she won’t and she (the midwife) was shouting. As I said, formally a relative will tell you that I went to this particular midwife and she gave me some slaps, but nowadays we have stopped all these things” (59 years, Nurse).

#### Birthing positions

Most health workers stated that women usually give birth in the lithotomy position (lying on the back with legs flexed), which was the preferred position of providers. Some of the health workers indicated that women’s birth positions are dependent on the discretion of the midwife who assists with the birth.

“Here we have only the lying, the lithotomy position, they lie down at the back and raise their legs” (36 years, Midwife)

“That one will go to the discretion of the midwife who is going to do the delivery if the position the woman prefers is not feasible in our setting or the midwife can’t get access to guide you to deliver, then the midwife will definitely not allow you but if it is feasible then the midwife can allow you” (27 years, Medical officer).

#### Birth companions

Most women were not allowed to have a birth companion, because health workers considered that labor ward environments were already over-crowded and unable to accommodate them. In such environments, providers described limited privacy for individual women—for example, multiple women laboring in the same room. Majority of the health workers indicated that women might be permitted a birth companion if she is the only patient in the labor ward, but this hardly ever occurs due to over-crowding.

“In our facility, how our first stage room is, there are so many patients so we cannot allow other people to come and see other people’ relatives, so we don’t allow any relative inside, unless you are alone in that room, if there are no patients around and you are alone, we can allow your relative to come and see you” (36 years, Midwife).

Some health workers indicated that some women prefer to have birth companions including their husbands. However, the labour rooms in most health facilities are not designed to accommodate the presence of birth companions due to multiple women in labor occupying the same and limited space.

“Yes, they would have wished, especially if their husband were around but you realize that this place is not like abroad” (57 years, Health Administrator)

#### Lack privacy for women during childbirth

Inadequate privacy for women during labour in health facilities was a major recurring theme that emerged from the health workers’ narratives. Lack of privacy was a major form of mistreatment and nearly all the health workers were emphatic about this deficiency or limitation in the health care delivery.

“Talking about privacy, it is not adequate for the women who comes to the hospital to deliver at the labor ward. For instance, in our first stage room we have six beds and they are all lined up so when there is the need to do something for the women, sometimes if you don’t have enough screens. There is no privacy that the woman would have” (51 years, Health Administrator).

### Perceived factors that influence mistreatment during childbirth

Health workers identified some factors contributing to mistreatment in their health facilities. These include lack of supplies, limited staff capacity, health facility policies and infrastructure and the women’s attitude.

#### Limited staff capacity

Participants generally agreed that there were inadequate numbers of staff on their wards resulting in overwhelming clinical workload. This led to additional stress on providers, affecting how they treated women. Staff become exhausted, irritable or frustrated, and consequently shout at women or ignore them if they are perceived as e uncooperative. This situation may be worsened when multiple women present at different stages of labor, or when only one or two midwives are on duty.

“Well, I think principally, I will say it is because of the workload. The number of midwives in this hospital basically is about 25 and you deliver…about 3,000 women in 6 months. So, the pressure of work is there. And at times you will come in the night and there is only one midwife on duty” (39 years, Hospital administrator).

#### Health facility policies and infrastructure

On the whole, health workers did not feel that the infrastructure influenced the occurrence of mistreatment. However, some doctors felt overworked by 24-h shifts, which negatively affected the quality of care they provided to the birthing women. The lack of a 24-h on-site pharmacy was also noted as a major challenge, as it required midwives to travel to obtain medications.

#### Perception of women’s non-compliance with instructions

According to the health workers, another major factor that influenced mistreatment was the behavior of the women. They believed that women were not compliant with instructions, inability to push during the second stage of labor and lack of co-operation sometimes provoke some of the health workers resulting in some forms of mistreatment.

“There are some patients who are called bad patients or difficult patients when you tell them do this, it’s always the opposite irrespective of how you communicated well to them or how well you are treating them, there are always bad nuts who will come and cause problems” (27 years, Medical officer).

“Basically, as a health worker you should tolerate all the attitudes that are thrown at you. But every human being will break at a point especially if you don’t get the cooperation of others” (31 years, Medical officer).

When women were viewed as non-compliant, then health workers justified the use of force as appropriate, rather than communication and encouragement. However, the preferences, needs and concerns of the women need to be incorporated or considered in this complex phenomenon of non-compliance to clinical instructions.

“She just wasn’t cooperative. I mean if you have a woman who wouldn’t let you listen to the fetal heart, you can imagine how frustrating it will be for you as a clinician because you are yearning to have a live baby and a well mother and you can’t monitor the fetal heart then you become a bit frustrated” (33 years, Medical officer).

### Acceptability or non-acceptability of different types of mistreatment

Most health workers stated that the acts of mistreatment are unacceptable and should not be practiced except when the act like shouting and beatings are used to prevent childbirth complications. Most participants strongly disagreed that pinching or slapping women was appropriate, although a few health workers together believed these acts were acceptable, if used to encourage women to cooperate.

“I don’t think under any normal circumstances should you slap or pinch someone during delivery. Because, we all know that labor is associated with pain and once someone is in pain she can say or do anything so you shouldn’t be provoked by the words she says or whatever she does. Yours is to encourage her to do whatever you need her to do” (26 years, Midwife).

“For pinching, I think it is okay because at times when you call their relatives, they will tell you that madam ‘wo nbo no nma me’ [madam won’t you beat her for me], the mother will come and be beating them” (37 years, Midwife).

Physically restraining the woman, holding her down, and shouting or yelling at the woman were also considered unacceptable by most health workers. They emphasized the need for health workers to speak calmly with the women since their seemingly unacceptable behaviors are expected because of the pain they might be going through. On the other hand, the minority argued that it could be acceptable to prevent adverse neonatal outcomes.

“In my opinion, I will say yes it can be acceptable—though it is not right—but it can be acceptable in order to prevent the mother from losing the baby. Because sometimes they might not know the seriousness in what they are doing or how far their actions may affect the results of the baby” (28 years, Midwife).

“At times the head will be stuck in the vagina; we asked them to push and they refuse by telling you they can’t push, meanwhile, we can’t do anything for you at that point. She will go blaming it on the midwife, saying the midwife killed her baby instead of blaming herself for not pushing, so you pinch her, with that pain she will push, so at times it’s acceptable (36 years, Midwife).

### Major challenges faced by health workers

Inadequate staffing, lack of equipment and logistics were identified by the health workers as the most challenging aspects of their work. Some indicated that excessive workload results in stressful situations and some experience severe headaches. Some respondents described situations when they become extremely frustrated and vulnerable because of unavailability of the needed equipment and logistics, and inadequate staff.

“Most challenging is when you come to work and you feel there are inadequate staff…like two staff against forty-five patients and you have emergency coming in, you feel stressed out, you get this headache, you feel very bad” (26 years, Midwife).

The study participants suggested that increasing the number of staff and health facilities, retraining of health workers and educating the women about labor and their what to expect could improve the quality of care. Regular short duration courses or workshops for the health workers was considered as an important step in minimizing the occurrence of mistreatment of women at health facilities.

“In my opinion, I think this place is already a friendly place for them, so what we can do to help is that we increase the number of staff working so that everybody can work happily without getting over worked and displacing their anger on any patient. Also, the patient should know what to expect before they even come to the hospital” (27 years, Medical officer).

“I will say in-service training is another important tool in the management of all sector health workers. So, in-service training first and then a sort of motivation for hard work…. you will reward hard work. Citations for the wards in general and then individuals who excel” (51 years, Health Administrator).

Also, motivation of the health workers for hard work was considered as a means on improving the work output in addition to provision of adequate logistics. Staff motivation may encourage the health workers to put up their best performance which may result in respectful maternity care.

“I think the basic thing that the health workers need from the hospital is availability of logistics and motivation. When I say motivation, it comes in various forms. Even getting up and going to the ward and just greeting them and encouraging them is enough motivation” (39 years, Health Administrator).

## Discussion

This paper gives important insights into health workers’ and hospital administrators’ perspectives about mistreatment of women during childbirth in health facilities in Ghana. The health workers reported mixed feelings regarding the quality of care experienced by women during childbirth. Generally, the participants were aware of the occurrence of mistreatment during childbirth, with specific mentions of physical abuse and verbal abuse. Common presentations of mistreatment reported by participants include slapping, forceful physical restraint, verbal abuse, denial of preferred birthing position and denial of birth companion. The major factors participants associated with mistreatment were limited staff capacity, high workloads and seeming perceptions of women not complying with clinical requests. There were mixed responses regarding the acceptability of mistreatment during childbirth although majority were against it. Increasing staff strength, number of health facilities, refresher training for health workers and adequate education of the women on what to expect in labour were proposed as measures to minimize mistreatment of women.

Previous literature showed that, women will consciously change their health facility and will not recommend it to others if they experience humiliating and intolerable behavior during childbirth [24]. Also, some women prefer to give birth at home because of the harsh treatment they receive from midwives at health facilities [6, 13, 25]. Generally, women are more likely to return for medical care in health facilities where health workers treat them well including respectful care [26]. On the other hand, some women might still access facilities they are not satisfied with simply because they have no alternatives. The perspectives of health workers in our study are consistent with the experiences of mistreatment described by women in Ghana in our previous research [6]. Surprisingly, some health workers do not consider these acts as mistreatment. They argue that, such actions serve to encourage cooperation and compliance from the women to enable favorable birth outcomes. This language around “women’s compliance” with clinical requests seems to place responsibility for poor treatment as the woman’s fault and aligns with other research on mistreatment against women. It also represents an important entry point for training providers on how to cope with stressful work environments and high workloads, while still providing woman-centered care.

Similarly, most of the women are usually denied their preferred birthing position [26–30] and compelled to deliver in the birth positions preferred by the midwives. Anecdotal information indicates that, the denial of women’s preferred birth positions, stems from the fact that the health workers lack expertise in conducting deliveries in other birthing positions apart from the supine or lithotomy position. More research is needed to explore how health workers in low resource settings can be trained to support women to give birth in more empowering positions of their choice.

In addition, birth companions can lead to better health and birth outcomes for women, including reduced rates of caesarean section, and better birth experiences [26, 28–32]. However, most women in Ghana do not have access to birth companionship. In this study, lack of privacy and birth companions during childbirth were attributed to the restrictive nature of the labour wards and high caseloads. In most instances, there are several labouring women in one labour room which precludes the needed privacy or birth companions. Thus, the labour wards may need redesigning or the use of curtains to provide privacy which may also allow for the presence of birth companions.

The challenge of mistreatment of women remains pervasive in the subregion and various manifestations of disrespectful care have been described such as verbal abuse, physical abuse, ineffective communication, discrimination and neglect [3, 33]. In South Africa, neglect is considered the most prevalent type of mistreatment and has been associated with inequality in healthcare access and utilization [34]. Persistence of this public health issue requires concerted regional and local interventions at multiple levels to address the menace including political will and appropriate institutional policies [35]. For instance, health workers in Kenya described the drivers of mistreatment of women including suboptimal supervision, demotivation and unavailability of relevant medical equipment and supplies [33]. Mistreatment of women during childbirth is considered a subset of violence against women which usually originates from structural gender inequality [35]. It is important to emphasize that mistreatment of women during childbirth is a longstanding obstetric phenomenon [36] and remains detrimental to achieving respectful maternity care [11]. Our study findings highlight the perpetuation of this obstetric violence in the subregion with a potential of disincentivizing women in seeking care at health facilities in their future maternities.

The persistence of mistreatment in health facilities and health system-related challenges, calls for improvement in the existing healthcare policies. Unsupportive working environment needs retooling to minimize intrinsic challenges such as inadequate staff number, high workload, unavailability of equipment/tools and long working hours. Training of staff and educating the women about labor and the expected outcomes are suggested recommendations to improve maternal care [37, 38]. Health facilities require re-designing to allow for birth companionship since their presence provide adequate psychological support to the laboring women.

### Strengths and limitations

The main strength of this study relates to it being part of a larger WHO multi-country research to develop consensus definition of mistreatment during childbirth and develop and validate tools for measuring disrespectful maternity care. Although the women’s perspectives on the subject have been published earlier, the corresponding views from the health workers are critical in preventing mistreatment and promoting respective maternity care. This study adequately utilized appropriate triangulation in the data collection (data source triangulation), transcription and analysis of the data. The use of research assistants experienced in qualitative interviews reduced the level of bias. To improve the validity of the findings, the issue of reflexivity was considered at various stages of the study including framing of the research question, data collection, analysis and interpretation via the inclusion multiple researchers of different but related medical fields [21].

The main limitation is that the study was conducted in only one geographical region of Ghana, and results may not be transferable to other settings. Also, the responses presented in this study do not include the actual experiences of mistreatment by the women themselves, which have been presented in a separate analysis [6]. Although this study has some recognizable limitations, the findings are significant collectively as evidence-base support in devising appropriate consensus-based criteria in measuring mistreatment of women during childbirth. Our research team included people with different training, expertise, and experiences, and these complementary backgrounds helped us to refine the research questions, design the methodology, and conduct thorough analysis. The convergence of multiple researchers with different reflexivity potentials strengthens the research, as we were able to both supplement and contest each other’s opinion.

## Conclusion

Women experience different forms of mistreatment during childbirth at health facilities with the common ones being physical and verbal abuse. The main rationale for mistreatment of women during childbirth, as perceived by the health workers, includes inadequate number of health professionals, unavailability of logistics, high patient load and non-compliance on the part of the women. Mistreatment of women was considered important in some clinical situation to prevent adverse birth outcomes and this indicates inadequate understanding of the clinical impact of mistreatment of women during childbirth. We recommend appropriate locally acceptable steps for integration and implementation of evidence-based measures to mitigate the burden of mistreatment in the country. Further research on implementation of evidence-based interventions to minimize mistreatment of women during childbirth is globally recommended to enhance positive intrapartum experience and respectful maternity care.

## Supplementary Information

**Additional file 1: Appendix 1**. In-depth interview guide for healthcare providers.

**Additional file 2: Appendix 2. **In-depth interview guide for hospital administrators.

## Data Availability

The full transcripts for this qualitative study are available upon request from the corresponding author.

## Abbreviations

COREQ: Consolidated Criteria for Reporting Qualitative Research
ERC: Ethical Review Committee
GHS: Ghana Health Service
HRP: Human Reproduction Programme
RMC: Respectful Maternity Care
WHO: World Health Organization
