# Supplementary material for: Health workers’ and hospital administrators’ perspectives on mistreatment of women during facility-based childbirth: a multicenter qualitative study in Ghana
Source: Reprod Health. 2022 Mar 29;19:82. doi: 10.1186/s12978-022-01372-3 (PMC8966263; doi:10.1186/s12978-022-01372-3)
Supplement: Supplementary file 1 — Additional file 1: Appendix 1. In-depth interview guide for healthcare providers. [file 12978_2022_1372_MOESM1_ESM.docx]

**Appendix 1: In-depth interview guide for healthcare providers**

**Healthcare providers In-depth interview guide**

**Step 1:** Introduce yourself to the participant. Describe the purpose of the interview and how information will be used. Obtain written consent.

**Step 2:**  Ask the participant to identify her/himself and fill out the table below on sociodemographic information prior to beginning the interview.

**Step 3:**  Conduct the interview. Please remember to audio record the interview.

**Step 4:** Complete the form at the end of the interview guide.

| **Participant #** | **Age group** (circle) | **Sex** (circle) | **Cadre and position**  (write in) | **Total years of service**  (circle) | **Years of service in current facility** (circle) |
| --- | --- | --- | --- | --- | --- |
| Participant | <30  30-45  >45 | Female  Male |  | < 1 year  1 – 5 years  > 5 years | < 1 year  1 – 5 years  > 5 years |

**Start time: : Interviewer ID __ __ __ __**

**Interview date: __ __/__ __/__ __**

DD / MM / YY

**Interview discussion guide**

A. Perceived decision-making process and expectations for women to deliver at a facility

*Please take a moment to think about your work on the labor and delivery ward and your patients.*

1. In your opinion, why do women seek care at facilities during delivery?
2. In your opinion, how is the decision made for women to seek care at facilities during delivery?
3. In your opinion, what do patients EXPECT when they seek care at the facility for childbirth? Please explain.
4. What do you think patients NEED when they seek care at the facility for childbirth? Please explain.

B. Elements of respectful care during childbirth

1. In your opinion, how are women treated by staff [administrators, nurses/midwives, physicians] in the facility when they come to deliver [from admission to discharge]?
   1. In your opinion, how should they be treated, if different than what you described?

C. Elements of disrespect and abuse to inform the development of the identification criteria, including involved parties, timing and frequency of disrespectful or abusive care

*Please take a moment to think about a time when a woman was mistreated or poorly treated by a coworker during labor and delivery.*

1. Could you tell me about this situation when a woman was mistreated by a coworker during labor and delivery.
   1. Explain the situation.
   2. Who was involved in the situation?
   3. How was the woman mistreated?
   4. When did it happen? [Probe: time of day, during labor, during delivery or postpartum].
   5. How often did it happen? [Probe: just once or more than often].

***** If the participant describes a situation related to either verbal mistreatment, physical mistreatment, or poor communication between the patient and provider, then probe the following accordingly:

| **Verbal mistreatment:** | **Physical mistreatment:** | **Poor communication between the patient and provider:** |
| --- | --- | --- |
| Did the provider raise his/her voice to the woman? | Did that include pinching? | Were there any problems with the language of communication? |
| What types of comments were made? | Did that include slapping? | If so, was a translator available? |
| Were these comments made to threaten the woman with poor outcomes? | Did that include beating? | Was the poor communication related to lack of consent for a test or procedure? |
| Were these comments judgmental/derogatory in nature? | Did that include kicking? |  |
| Were these comments based on the woman's age or her number of children? | Did that include hitting? |  |
| Did the provider place blame on the woman for getting pregnant? |  |  |

1. In your opinion, how common is the situation that you described? [Probe: do situations like this happen often?]

*Now I would like to ask your opinion on the treatment of women during labor and delivery.*

1. Are there any situations where it would be acceptable for a provider to pinch or slap a woman during delivery? Please explain.
2. Are there any situations where it would be acceptable for a provider to not ask for the patient’s consent before a test or procedure when the patient is conscious? Please explain.
3. Are there any situations where it would be acceptable for a provider to tell the woman she will have a poor outcome if she does not cooperate? Please explain.
4. In your opinion, what could be done to improve the treatment of women during labor and delivery?

D. Perceived factors that influence disrespect and abuse in the facilities

1. In your opinion, what are the factors that influence the mistreatment of women during labor and delivery? Please explain. Probe:
   1. Related to supplies (availability of medication, equipment)
   2. Related to health provider staffing (number of staff, attitude towards patients)
   3. Related to patient load (number of patients, overcrowding)
   4. Related to your health facility (policies, infrastructure, services)
2. In your opinion, what could be done to address these factors so that women are treated better during labor and delivery?

E. How staff are treated (*remind participant that all responses will be confidential and their responses will not impact their job in any way).*

1. Do you have a mentor at work? (Probe: if yes, ask participant to elaborate on the relationship with the mentor. If no, ask if there is anyone at work who they go to for work-related advice?).
2. Could you please describe for me what the relationship that you have with your supervisor is like? (Probe: what is the job title of your supervisor?)
3. Do you feel that your supervisor supports you in your work responsibilities? Please explain (probe: could you tell me about a time when your supervisor supported you? Could you tell me about a time when your supervisor did not support you?
4. Could you please describe for me what the relationship that you have with your peers is like? (Probe: do you feel supported by your peers? If you are struggling to meet the demands of your work, can you look to your peers for help?).
5. Overall, do you feel that your work environment is supportive? Please explain.
6. How do you feel your training prepared you for your current position?

**When the interview appears to have finished,** ask participant if there is anything that you have misunderstood or that they would like to add.

Thank the participant for his/her time. Remind them that the information will be kept confidential.

**End time :**
