# Supplementary material for: Health workers’ and hospital administrators’ perspectives on mistreatment of women during facility-based childbirth: a multicenter qualitative study in Ghana
Source: Reprod Health. 2022 Mar 29;19:82. doi: 10.1186/s12978-022-01372-3 (PMC8966263; doi:10.1186/s12978-022-01372-3)
Supplement: Supplementary file 2 — Additional file 2: Appendix 2. In-depth interview guide for hospital administrators. [file 12978_2022_1372_MOESM2_ESM.docx]

**Appendix 2: In-depth interview guide for hospital administrators**

**Hospital administrators In-depth interview guide**

**Step 1:** Introduce yourself to the participant. Describe the purpose of the interview and how information will be used. Obtain written consent.

**Step 2:**  Ask the participant to identify her/himself and fill out the table below on sociodemographic information prior to beginning the interview.

**Step 3:**  Conduct the interview. Please remember to audio record the interview.

**Step 4:** Complete the form at the end of the interview guide.

| **Participant #** | **Age group** (circle) | **Sex** (circle) | **Position**  (write in) | **Total years of service**  (circle) | **Years of service in current facility** (circle) |
| --- | --- | --- | --- | --- | --- |
| Participant | <30  30-45  >45 | Female  Male |  | < 1 year  1 – 5 years  > 5 years | < 1 year  1 – 5 years  > 5 years |

**Start time: : Interviewer ID __ __ __ __**

**Interview date: __ __/__ __/__ __**

DD / MM / YY

**Key informant interview guide**

A. Perceived factors that influence disrespect and abuse in the facilities

*Sometimes women are mistreated during childbirth by providers and staff at health facilities. This mistreatment may take several different forms. I would like to discuss the factors that influence this type of mistreatment with you.*

1. In your opinion, what are the factors that influence the mistreatment of women during labor and delivery? Please explain. Probe:
   1. Related to supplies (availability of medication, equipment)
   2. Related to health provider staffing (number of staff, attitude towards patients)
   3. Related to patient load (number of patients, overcrowding)
   4. Related to policies in your facility
   5. Other factors at a health facility level
   6. Other factors at a health system level
2. What could be done to address these factors so that women are treated better during labor and delivery?
   1. From a facility administration perspective?
   2. From a larger health system perspective?

B. How staff are treated (*remind participant that all responses will be confidential and their responses will not impact their job in any way).*

1. Do you have a mentor at work? (Probe: if yes, ask participant to elaborate on the relationship with the mentor. If no, ask if there is anyone at work who they go to for work-related advice?).
2. Could you please describe for me what the relationship that you have with your supervisor is like? (Probe: what is the job title of your supervisor?)
3. Do you feel that your supervisor supports you in your work responsibilities? Please explain (probe: could you tell me about a time when your supervisor supported you? Could you tell me about a time when your supervisor did not support you?
4. Could you please describe for me what the relationship that you have with the doctors, nurses and midwives in your facility is like?
5. Overall, do you feel that your work environment is supportive? Please explain.
6. How do you feel your training prepared you for your current position?

**When the interview appears to have finished,** ask participant if there is anything that you have misunderstood or that they would like to add.

Thank the participant for his/her time. Remind them that the information will be kept confidential.

**End time :**
